# A novel high mobility group box 1 neutralizing chimeric antibody attenuates drug‐induced liver injury and postinjury inflammation in mice

**DOI:** 10.1002/hep.28736

**Published:** 2016-09-01

**Authors:** Peter Lundbäck, Jonathan D. Lea, Agnieszka Sowinska, Lars Ottosson, Camilla Melin Fürst, Johanna Steen, Cecilia Aulin, Joanna I. Clarke, Anja Kipar, Lena Klevenvall, Huan Yang, Karin Palmblad, B. Kevin Park, Kevin J. Tracey, Anna M. Blom, Ulf Andersson, Daniel J. Antoine, Helena Erlandsson Harris

**Affiliations:** ^1^ Department of Medicine, Rheumatology UnitKarolinska InstituteStockholmSweden; ^2^ MRC Centre for Drug Safety Science, Department of Molecular & Clinical PharmacologyLiverpool UniversityLiverpoolUnited Kingdom; ^3^ Department of Women's and Children's HealthKarolinska InstituteStockholmSweden; ^4^ Section of Medical Protein Chemistry, Department of Translational MedicineLund UniversityMalmöSweden; ^5^ Laboratory of Biomedical ScienceThe Feinstein Institute for Medical ResearchManhassetNYUSA

## Abstract

Acetaminophen (APAP) overdoses are of major clinical concern. Growing evidence underlines a pathogenic contribution of sterile postinjury inflammation in APAP‐induced acute liver injury (APAP‐ALI) and justifies development of anti‐inflammatory therapies with therapeutic efficacy beyond the therapeutic window of the only current treatment option, *N*‐acetylcysteine (NAC). The inflammatory mediator, high mobility group box 1 (HMGB1), is a key regulator of a range of liver injury conditions and is elevated in clinical and preclinical APAP‐ALI. The anti‐HMGB1 antibody (m2G7) is therapeutically beneficial in multiple inflammatory conditions, and anti‐HMGB1 polyclonal antibody treatment improves survival in a model of APAP‐ALI. Herein, we developed and investigated the therapeutic efficacy of a partly humanized anti‐HMGB1 monoclonal antibody (mAb; h2G7) and identified its mechanism of action in preclinical APAP‐ALI. The mouse anti‐HMGB1 mAb (m2G7) was partly humanized (h2G7) by merging variable domains of m2G7 with human antibody‐Fc backbones. Effector function‐deficient variants of h2G7 were assessed in comparison with h2G7 *in vitro* and in preclinical APAP‐ALI. h2G7 retained identical antigen specificity and comparable affinity as m2G7. 2G7 treatments significantly attenuated APAP‐induced serum elevations of alanine aminotransferase and microRNA‐122 and completely abrogated markers of APAP‐induced inflammation (tumor necrosis factor, monocyte chemoattractant protein 1, and chemokine [C‐X‐C motif] ligand 1) with prolonged therapeutic efficacy as compared to NAC. Removal of complement and/or Fc receptor binding did not affect h2G7 efficacy. *Conclusion:* This is the first report describing the generation of a partly humanized HMGB1‐neutralizing antibody with validated therapeutic efficacy and with a prolonged therapeutic window, as compared to NAC, in APAP‐ALI. The therapeutic effect was mediated by HMGB1 neutralization and attenuation of postinjury inflammation. These results represent important progress toward clinical implementation of HMGB1‐specific therapy as a means to treat APAP‐ALI and other inflammatory conditions. (Hepatology 2016;64:1699‐1710).

**Abbreviations**
ALD: alcoholic liver disease
ALF: acute liver failure
ALI: acute liver injury
ALT: alanine aminotransferase
ANOVA: analysis of variance
APAP: acetaminophen
APAP‐ALI: acetaminophen‐induced acute liver injury
CBA: cytometric bead array
CXCL: chemokine (C‐X‐C motif) ligand
DILI: drug‐induced liver injury
ELISA: enzyme‐linked immunosorbent assay
endoS: endoglycosidase‐S
FcγR: Fcγ receptors
GSH: glutathione
HMGB1: high mobility group box 1
Ig: immunoglobulin
IP: intraperitoneal
I/R: ischemia‐reperfusion
LCA: *Lens culinaris* agglutinin
LT: liver transplantation
mAb: monoclonal antibody
MCP‐1: monocyte chemoattractant protein 1
MD‐2: myeloid differentiation protein 2
miR‐122: microRNA‐122
NAC: *N*‐acetylcysteine
NHS: normal human serum
PBS: phosphate‐buffered saline
SDS‐PAGE: sodium dodecyl sulfate polyacrylamide gel electrophoresis
SPR: surface plasmon resonance
TLR: Toll‐like receptor
TNF: tumor necrosis factor

Drug‐induced liver injury (DILI) is the leading cause of acute liver injury (ALI), which is both a clinical concern and results in drug attrition, issuing of black box warnings, and drug withdrawal from the market.^1^ Acetaminophen (APAP) is an analgesic and antipyretic and is generally safe when taken at a therapeutic dose; however, intentional or unintentional overdoses can lead to APAP‐induced acute liver injury (APAP‐ALI) and represent around 50% of all cases of acute liver failure (ALF).^2^, ^3^ Clinical APAP intoxication is counteracted by peroral or intravenous administration of *N*‐acetylcysteine (NAC), which has a narrow window for therapeutic intervention. Untreated, APAP overdoses can lead to fulminant liver failure, and the clinical outcome ranges from full recovery, a need of liver transplantation (LT), or even death. The post‐APAP‐associated inflammation (especially innate immune activation), resulting from a substantial hepatocellular necrosis, is associated with poor clinical outcome (i.e., increased need for LT or death). For late presenting patients, in whom inflammatory responses are fulminant,^4^ there are no effective treatment options for severe cases of ALF beside LT. Because of the shortage of potential liver donors and cost of LTs, there is an urgent need for development of alternative therapeutic strategies with broader therapeutic time windows than NAC, including immunomodulatory drugs, in preventing APAP‐ALI progression.

High mobility group box 1 (HMGB1) was discovered 16 years ago as an endogenous inflammatory mediator and now serves as a prototype for the class of proinflammatory mediators denoted Alarmins. Alarmins are released passively during cell death or from stressed cells. During infectious and sterile inflammatory conditions, HMGB1 is also actively released by immune cells, enhancing and perpetuating inflammation and thus contributing to the pathogenesis of a great number of inflammatory diseases. The diverse inflammatory functions of HMGB1 are mediated by multiple, different reciprocal receptors. Global structural changes associated with cysteine redox modifications of HMGB1 control its receptor usage and thereby its bioactivities. HMGB1 induces cell migration in its fully reduced, all‐thiol form when interacting with chemokine (C‐X‐C motif) ligand (CXCL)‐12 and C‐X‐C chemokine receptor type 4,^5^ whereas cytokine production is induced by the disulfide form interacting with the myeloid differentiation protein 2 (MD‐2)/Toll‐like receptor (TLR) 4 receptor complex.^6^, ^7^ Furthermore, HMGB1 has also been demonstrated to signal through receptor for advanced glycation end products, TLR2, TLR9, CD24/Siglec, and T‐cell immunoglobulin and mucin‐domain containing‐3,^8^ with undetermined requirements for possible posttranslational modifications. Additionally, active secretion of HMGB1 is regulated by acetylation of HMGB1,^9^ an HMGB1‐specific modification that negatively correlates with APAP‐ALI patient outcome.^10^

HMGB1 plays a critical role in a wide array of liver disease conditions, including liver ischemia‐reperfusion (I/R) injury, alcoholic liver disease (ALD), cholestasis, and DILI. In liver I/R injury, necrotic cell death is prominent and HMGB1 is released.^11^ The pathogenic contribution of HMGB1 in hepatic I/R has recently been shown through blockade of the interaction between HMGB1 and MD‐2.^7^ Acetylated HMGB1 has been recorded in ALD patients and ethanol‐fed mice, demonstrating an inflammatory component and active HMGB1 release in ALD. Furthermore, conditional hepatocyte ablation of *Hmgb1* is protective in a mouse model of ethanol‐induced liver injury.^12^ Similar HMGB1 isoforms have been recorded in obstructive cholestasis patients,^13^ supporting an active release and inflammatory role of HMGB1 in this disease as well. HMGB1 is required for post‐APAP injury inflammation and has been shown to be pivotal in the progression of APAP‐ALI, and hepatocyte‐specific HMGB1 deficiency improves survival.^14^ In a clinical setting, HMGB1 serves as a promising sensitive and specific biomarker of APAP‐ALI, outperforming alanine aminotransferase (ALT) as a marker of progression and as an indicator of outcome.^2^, ^10^ The initial APAP‐induced hepatocyte necrosis results in an initial release of all‐thiol HMGB1. This leads to recruitment and activation of immune cells, which propagate the inflammatory response, resulting in increased hepatocyte death and exacerbation of injury.^14^ HMGB1‐specific antibody treatments have consolidated the pathogenic contribution of HMGB1 in APAP‐ALI, demonstrating increased survival.^15^

Therapies targeting either the release of HMGB1, interfering with HMGB1‐receptor signaling or directly antagonizing HMGB1 (i.e., box A therapy), ameliorate disease severity and promote survival in a wide spectrum of experimental disease models.^16^ These therapies are, however, unspecific in the sense that they may affect other ligand‐receptor interactions or signaling pathways utilized by other molecules than HMGB1. They may thus not be suitable for clinical use. Importantly, targeting HMGB1 with the use of antibodies specifically affects extracellular HMGB1 bioactivities, but will not interfere with its intracellular functions. Successful HMGB1‐specific polyclonal antibody therapy was first described in an acute inflammatory model of sepsis^17^ and later in a chronic setting of experimental arthritis models.^18^ Polyclonal and monoclonal antibody (mAb)‐based therapies are powerful tools in preclinical research. However, long‐term clinical success in humans with such antibodies is hampered by the inherent immunogenicity of xenogeneic antibodies that may cause safety issues and a negative impact on clinical efficacy.^19^ The development of humanized antibodies has significantly reduced the restricting xenogeneic immune responses. Chimeric antibodies with the antigen‐binding region kept xenogenic, targeting self‐antigens are presently used successfully to treat cancer (anti‐CD20/rituximab), graft‐versus‐host disease (anti‐CD25/basiliximab), and various autoimmune diseases (anti‐TNF [tumor necrosis factor]/infliximab). The heterogeneity of diseases or disorders with an inflammatory component emphasizes a continuous search for treatment refinement and creation of future therapies that specifically targets novel pathogenic molecules.

To enable development of HMGB1‐targeted therapy for clinical use, we set out to engineer a chimeric anti‐HMGB1 mAb (h2G7) by preserving the variable regions of an extensively studied and effective mouse mAb (m2G7) with recorded beneficial anti‐inflammatory effects in multiple preclinical models (Supporting Table S1). To verify well‐maintained beneficial therapeutic effects, we utilized a highly HMGB1‐dependent experimental model of APAP‐ALI, which established that h2G7 provided equal therapeutic benefit as its murine analog. By modification of the CH2 domain, we could generate a variant of h2G7 unable to activate the classical complement pathway (K322A mutant) and an h2G7 variant incapable of binding Fc‐receptors (endoglycosidase‐S [endoS]‐treated h2G7). By comparing the therapeutic *in vivo* efficacy of these three mAb variants, we conclude that h2G7 treatment alleviated APAP‐ALI through HMGB1 neutralization and has a prolonged therapeutic window, as compared to NAC treatment.

## Materials and Methods

A detailed description of experiments is described in the Supporting Methods.

A chimeric anti‐HMGB1 antibody (h2G7) with human immunoglobulin (Ig) G1 isotype was generated as described.^20^, ^21^ Briefly, cDNA encoding the 2G7 mouse variable immunoglobulin domains was polymerase chain reaction amplified (Supporting Table S2) and subcloned into plasmids encoding human constant domains. Antibody specificity was tested by coating plates with HMGB1, box A, or box B followed by titration with increasing concentrations of mAbs. Affinities were analyzed by surface plasmon resonance (SPR). Briefly, mAbs were immobilized on a CM5‐dextran chip and recombinant HMGB1 was injected at various concentrations (0, 55, 110, 220, and 880 nM). Determination of dissociation constants was performed using Langmuir binding.

Male C57BL/6J or CD‐1 mice (Charles River, Margate, UK) were fasted (15‐16 hours) before intraperitoneal (IP) injection of APAP (530 or 300 mg/kg, when indicated; Supporting Fig. S1). At 2 hours post‐APAP (or 6 hours, when indicated), 300 μg of anti‐HMGB1 antibodies, box A, E2 (irrelevant isotype control), or 500 mg/kg of NAC were injected IP. At 10 hours post‐APAP (or 24 hours, when indicated), liver injury was determined by histology, serum ALT, and expression of microRNA‐122 (miR‐122). Inflammatory mediators were analyzed by cytometric bead array (CBA; BD Bioscience, Stockholm, Sweden).

Effector function‐deficient variants of h2G7, incapable of binding complement or Fc receptors, were generated by site‐directed mutagenesis (K322A; Supporting Table S2) or by endoS treatment. Inability to activate complement was validated by a decrease in C1q deposition from normal human serum (NHS). Antibody deglycosylation was validated by a characteristic mass‐shift (sodium dodecyl sulfate polyacrylamide gel electrophoresis [SDS‐PAGE] analysis) and *Lens culinaris* agglutinin (LCA) binding. Briefly, antibodies were coated onto plates followed by addition of biotinylated LCA and subsequently visualized with streptavidin/horseradish peroxidase and 3,3′,5,5′‐tetramethylbenzidine. Fcγ receptor (FcγR) binding was performed by binding of antibodies to human CD64‐coated plates or to live THP‐1 cells. For binding to THP‐1 cells, h2G7 variant antibodies were incubated with cells followed by detection with a fluorescein isothiocyanate‐conjugated F(ab′)2 anti‐human antibody.

### STATISTICAL ANALYSIS

Statistical analysis for *in vivo* studies was performed with Kruskal‐Wallis (Dunn's posttest), and for *in vitro* data, one‐way analysis of variance (ANOVA) was performed, where indicated. All statistical analysis was performed using GraphPad Prism.

## Results

### CHARACTERIZATION OF CHIMERIC 2G7 mAb BIOACTIVITIES

**Figure 1:**
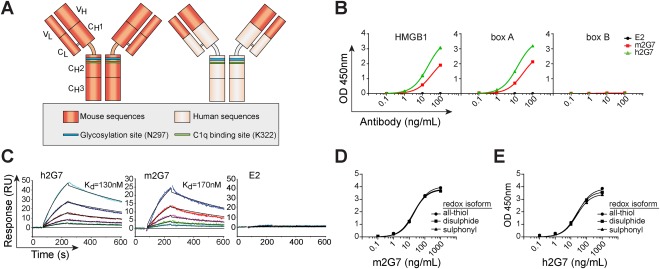
Chimeric anti‐HMGB1 antibody has retained specificity and affinity as its murine analog. (A) Constant and variable domain comparison of the parental mAb m2G7 and the chimeric h2G7 antibody denoted with known key residues regulating antibody effector functions. (B) Antigen specificity was tested by direct ELISA. HMGB1 (left), box A (middle), or box B (right) coated plates were incubated with antibodies at increasing concentrations. h2G7 (green) and m2G7 (red) displayed similar antigen specificity by binding to both HMGB1 and the box A domain, but not to box B. The control human IgG1 antibody E2 (black) did not display binding to any of the proteins. (C) SPR analysis was performed to define binding affinities to HMGB1. Antibodies were immobilized on a CM5‐dextran chip, and HMGB1 was injected at various concentrations (55, 100, 220, 440, or 88 0nM). h2G7 displayed slightly higher affinity (left, K_d_ = 130 nM) toward HMGB1, as compared to m2G7 (middle, K_d_ = 170 nM). No signal was detected from the E2 channel (right). (D,E) Redox isoforms of HMGB1 were coated and (D) m2G7 or (E) h2G7 were added at increasing concentrations and detected with anti‐mouse IgG or anti‐human IgG, respectively. Abbreviations: OD, optical density; RU, response units.

We merged the variable domains of 2G7, obtained from mouse (m2G7) hybridoma cells, with plasmids encoding the human IgGγ1 isotype and IgGκ backbone in order to generate a chimeric anti‐HMGB1 antibody (h2G7; Fig. 1A). The specific parent anti‐HMGB1 antibody (m2G7), that does not recognize HMGB2, binds to the amino acids 53‐63 within the box A domain of rodent and human HMGB1.^22^ Given that antibody specificity may be influenced by a change of IgG isotype,^23^ we examined the antigen specificity of h2G7 compared to m2G7. In similarity to m2G7, h2G7 displayed dose‐dependent binding to full‐length HMGB1 and the box A domain, but not to the box B domain (Fig. 1B). By utilizing SPR, we defined the antibody binding affinities for m2G7 (K_d_ 170 nM) and h2G7 (K_d_ 130 nM; Fig. 1C). This suggests that h2G7 had a slightly higher affinity toward HMGB1 as compared to its murine counterpart, although the recorded affinities were within the same order of magnitude. The human irrelevant IgG1 isotype control (E2)^20^ did not bind to HMGB1, or the box A or box B domains (Fig. 1B,C). Distinct functional HMGB1 cysteine redox isoforms are present during different phases of APAP‐induced inflammation,^6^, ^15^ and it was previously unknown whether 2G7 selectively binds to any of these redox isoforms. We therefore evaluated the binding capacity of m2G7 (Fig. 1D) and h2G7 (Fig. 1E) to all‐thiol, disulfide, and sulphonyl HMGB1. Both m2G7 and h2G7 displayed equal binding to all three isoforms tested, indicating that 2G7 has the capacity to antagonize HMGB1 during separate stages of inflammation.

### EQUIVALENT THERAPEUTIC EFFECTS OF h2G7 AND m2G7

**Figure 2:**
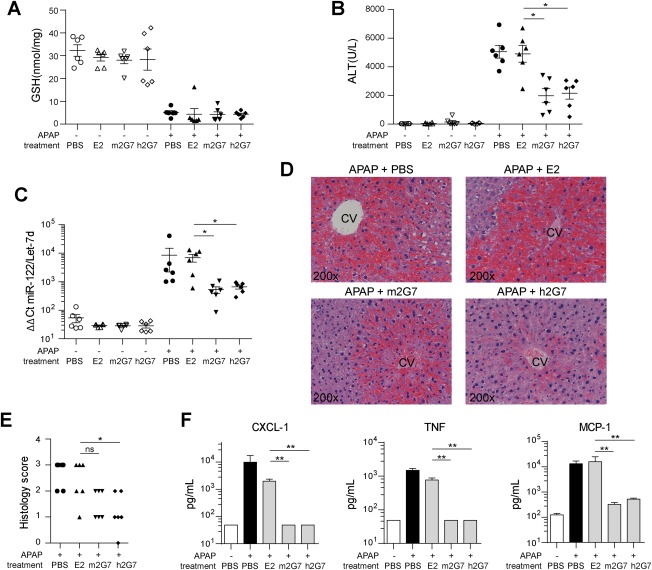
Similar therapeutic efficacy of h2G7 and m2G7 following APAP‐ALI. Fasted C57BL/6J mice were subjected to 530 mg/kg of APAP. PBS, 300 μg of m2G7, h2G7, or IgG1 control antibody (E2) were administered at 2 hours post‐APAP (n = 6). At 10 hours post‐APAP, (A) hepatic GSH, (B) serum ALT, (C) serum miR‐122 expression, (D) representative histological sections (hematoxylin and eosin stainings), and (E) histological score were analyzed to evaluate hepatic injury. Levels of the inflammatory mediators (F), TNF, CXCL‐1, and MCP‐1, in serum were quantified. If undetectable, values were substituted with the lowest limit of detection (50 pg/mL). Data are presented as means ± SEM. ^*^ *P* < 0.05 and ^**^ *P* < 0.01 by Kruskal‐Wallis with Dunn's posttest. Abbreviation: Ct, threshold cycle.

The therapeutic *in vivo* effects of h2G7 were evaluated in an experimental model of APAP‐induced ALI (Supporting Fig. S1). The pathogenic importance of HMGB1 in this model has previously been established by several reports.^2^, ^7^, ^15^, ^24^, ^25^ Non‐APAP‐challenged C57BL/6J mice were treated with phosphate‐buffered saline (PBS; vehicle), h2G7, m2G7, or the control E2 antibody. None of the treatments significantly affected hepatic glutathione (GSH) levels, as compared to the PBS control, indicating that the treatments alone did not affect hepatic APAP metabolite‐scavenging ability or APAP‐bioconversion (Fig. 2A). No difference was recorded between the treatment groups in non‐APAP‐challenged mice with respect to serum ALT or miR‐122, a sensitive biomarker for hepatocyte damage (Fig. 2B,C). These results indicate that antibody treatments were neither hepatotoxic nor did they alter baseline levels of systemic liver injury markers.

In mice challenged with APAP for 10 hours, hepatic GSH concentrations dropped from 32.4 ± 6.2 to 5.1 ± 1.9 nmols/mg ± SD (Fig. 2A). A comparable GSH depletion was observed in all mice treated with E2, m2G7, and h2G7 at 2 hours post‐APAP, indicating similar APAP metabolism between treatment groups (Fig. 2A). In concordance with previous studies based on antibodies targeting HMGB1 in APAP‐challenged mice,^7^, ^15^ 2G7 treatments mediated a significant reduction in serum ALT and miR‐122 levels compared to the E2 control group (Fig. 2B,C). No significant difference was observed between the m2G7 and h2G7 treatment groups, indicating equivalent hepatoprotective properties (Fig. 2B,C). APAP‐challenged mice showed characteristic hepatic pathophysiological changes, as compared to normal mice (Fig 2D and Supporting Fig. S2), and hepatic tissue expression of HMGB1 was lost in central necrotic areas, but up‐regulated in periportal areas (Supporting Fig. S2). Confirmatory histological results revealed reduced tissue destruction and alleviated inflammation in h2G7‐treated mice (Fig. 2D,E). A clear trend in hepatoprotection, as analyzed by histology, was also observed with m2G7 treatment, although this difference was not statistically significant (Fig. 2E). Ki‐67 stainings revealed that both m2G7 and h2G7 treatment had significantly less proliferating hepatocytes, as compared to livers of E2‐treated mice (Supporting Fig. S3), suggesting a decrease in liver regeneration at 10 hours post‐APAP. However, this may be explained by the decrease in liver injury observed in m2G7‐ and h2G7‐treated mice (Fig. 2B‐E).

Inflammatory mediators are up‐regulated as a result of the initial toxic hepatocellular injury induced by APAP exposure.^26^, ^27^ Both PBS‐ and E2‐treated mice demonstrated a significant increase in serum levels of monocyte chemoattractant protein 1 (MCP‐1), CXCL‐1, and TNF in APAP‐exposed mice compared to non‐APAP‐challenged animals (Fig. 2F). Anti‐HMGB1 treatments significantly reduced APAP‐induced serum levels of MCP‐1 and completely abolished the serum levels of CXCL‐1 and TNF, as compared to the E2 treatment group. Both interleukin‐1β and interferon‐γ have been reported to be up‐regulated post‐APAP administration^27^; however, these cytokines were undetectable in our experiments (data not shown).

**Figure 3:**
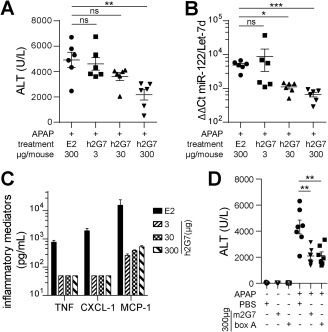
Dose‐dependent hepatoprotection and completely abolished inflammation with anti‐HMGB1 therapy. (A‐C) APAP‐challenged C57BL/6J mice (530 mg/kg) were treated (2 hours post‐APAP) with h2G7 (3, 30, and 300 μg/mouse) or with 300 μg of E2 control antibody (n = 6). Serum levels of (A) ALT and (B) miR‐122 indicate a dose‐dependent hepatoprotection with h2G7 as treatment and a complete abrogation of (C) inflammatory mediators, independent of therapeutic dose. If undetectable, values were substituted with the lowest limit of detection (50 pg/mL). (D) Serum ALT levels in APAP‐challenged CD‐1 mice (530 mg/kg) treated (2 hours post‐APAP) with PBS, 300 μg of m2G7, or 300 μg of box A for 10 hours (n = 5 or 7). Results are presented as means ± SEM. ^*^ *P* < 0.05; ^**^ *P* < 0.01; and ^***^ *P* < 0.001 by Kruskal‐Wallis with Dunn's posttest. Abbreviations: Ct, threshold cycle; ns, not significant.

To evaluate the lowest effective therapeutic dose, we administered one and two orders of magnitude less of h2G7 antibody (i.e., 300, 30, or 3 μg/mouse). A dose‐dependent decrease in serum ALT and miR‐122 (Fig. 3A,B) was observed. Interestingly, the lowest dose of h2G7 (3 μg) did not significantly decrease serum levels of ALT or miR‐122, but were still able to significantly block APAP‐induced inflammation (Fig. 3C). This supports that biomarker levels of hepatic injury is a combined consequence of direct APAP‐mediated toxicity and subsequent inflammation.

In order to explore whether the recorded hepatoprotection of 2G7 treatment was an effect observed exclusively in inbred mice, we also investigated the therapeutic effect of m2G7 treatment in an outbred CD‐1 mouse strain. In agreement with the results observed in C57BL/6J mice, m2G7‐treated CD‐1 animals expressed significantly reduced serum levels of ALT, as compared to PBS‐treated animals (Fig. 3D). HMGB1‐induced inflammatory effects are known to be antagonized by the truncated box A domain of the HMGB1 molecule, by means that are not fully resolved. These therapeutic results have previously been demonstrated in multiple experimental disease models,^18^, ^28^, ^29^ but never before studied in APAP‐ALI. A single IP injection of box A showed similar hepatoprotective effects as the m2G7 treatment (Fig. 3D), confirming that other strategies for extracellular HMGB1 blockade are also beneficial in APAP‐ALI. Injection of box A or m2G7, in non‐APAP‐challenged CD‐1 mice, did not alter baseline hepatic GSH or serum ALT levels (Fig. 3D and Supporting Fig. S4). In line with these observations, a uniform decrease of hepatic GSH was observed in all APAP challenged CD‐1 mice regardless of treatment (Supporting Fig. S4).

### SPECIFIC ANTI‐HMGB1 THERAPY HAS A DELAYED THERAPEUTIC WINDOW AS COMPARED TO NAC

**Figure 4:**
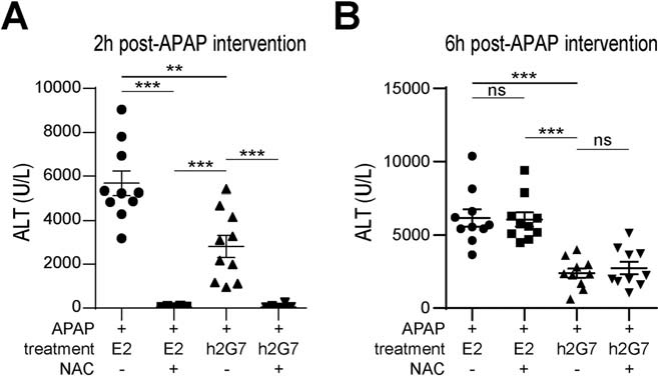
Targeting postinjury inflammation with h2G7 prolongs the therapeutic window of opportunity compared to NAC treatment. APAP‐challenged C57BL/6J mice (300 mg/kg) were treated (A) 2 or (B) 6 hours post‐APAP with 300 μg of h2G7, 300 μg of E2 control antibody or 500 mg/kg of NAC alone or in combination (n = 10) and sacrificed at 24 hours post‐APAP. Hepatoprotection was recorded as a decrease in serum ALT levels. Data are presented as means ± SEM. ^*^ *P* < 0.05; ^**^ *P* < 0.01; and ^***^ *P* < 0.001 by Kruskal‐Wallis with Dunn's posttest. Abbreviations: ns, not significant.

Postinjury inflammation is highly deleterious in APAP‐ALI patients and is especially evident in late‐presenting patients where NAC treatment fails to confer hepatoprotection. We therefore wanted to investigate whether anti‐HMGB1 treatment provided an extended window of therapeutic intervention. APAP‐challenged mice (300 mg/kg) were treated with either NAC, E2, or h2G7 at 2 or 6 hours post‐APAP and monitored for 24 hours. In line with previous reports, NAC treatment at 2 hours post‐APAP completely abrogated the increase in serum ALT (Fig. 4A). However, NAC failed to confer hepatoprotection at 6 hours post‐APAP, whereas h2G7 treatment was hepatoprotective at both 2 and 6 hours post‐APAP, as measured by serum ALT levels (Fig. 4A,B). The reality in the clinic is that most APAP‐ALI patients receive NAC treatment, and we thus, in addition, investigated the effect of NAC‐h2G7 combination treatment. We could not record any beneficial effects of the combination treatments at any time point (Fig. 4A,B), supporting that h2G7 treatment rather targets the postinjury inflammation than the initial metabolic injury that is counteracted by NAC treatment.

### GENERATION OF EFFECTOR FUNCTION‐DEFICIENT h2G7 VARIANTS

**Figure 5:**
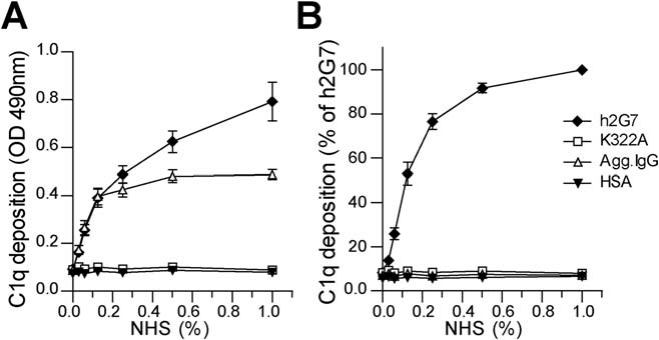
Abrogated binding to complement C1q *in vitro* by a K322A mutation. Plates were coated with indicated antibodies directly or with HMGB1. (A) Deposition of C1q from normal human sera (NHS) on plates coated with K322A or h2G7. Human serum albumin (HSA) or aggregated human IgG (Agg.IgG) were used as negative and positive controls, respectively. (B) K322A did not bind to C1q when complexed with HMGB1. In experiments with HMGB1 coating, data were normalized toward the h2G7 signal at 1% NHS, which was set as 100% C1q deposition. Results are represented as means ± SEM from three independent experiments. Abbreviation: OD, optical density.

Immunomodulatory effects of antibody therapies can be mediated by several different mechanisms. Antibodies may block the function of an antigen by binding and neutralizing its target, by activating the classical complement pathway through interaction with C1q or by the engagement of FcγRs and thus inducing cell‐mediated effects. In order to outline the therapeutic importance of Fc‐mediated effector functions of h2G7 antibody in APAP‐ALI, we generated effector function‐deficient variants by modifying its CH2 domain either by site‐directed mutagenesis or by endoS treatment, which removes N‐linked glycosylation on the Fc part of antibodies.^30^, ^31^, ^32^ Importantly, none of the Fc‐modified variants of h2G7 affected the binding to HMGB1 (data not shown). A lysine‐to‐alanine mutant variant of h2G7 was generated (K322A), and in contrast to nonmodified h2G7, the K322A variant completely abolished binding to human C1q *in vitro* when coated directly (Fig. 5A) or when bound to plates coated with HMGB1 (Fig. 5B). In similarity to the K322A mutant, endoS‐treated h2G7 did not bind to C1q (data not shown).

**Figure 6:**
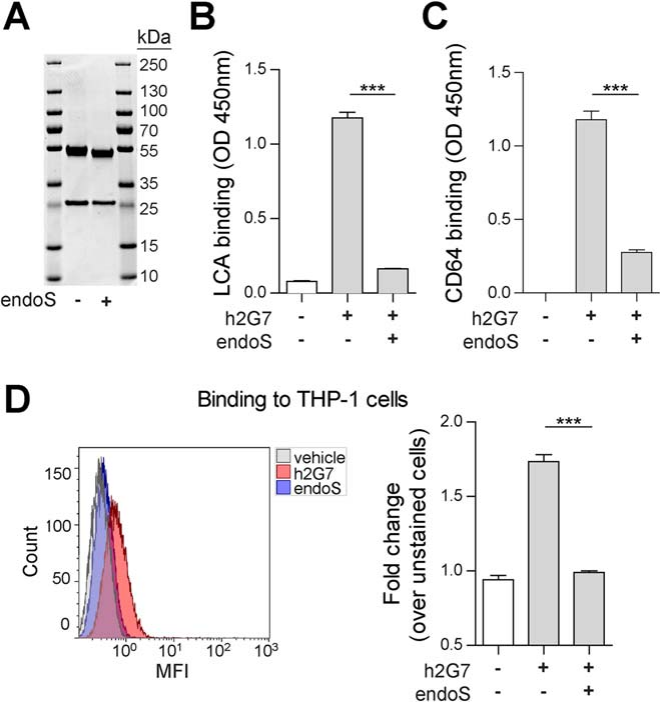
Aglycosylated h2G7 display reduced binding to Fc receptors *in vitro*. Deglycosylation of h2G7 was performed by endoS treatment and was analyzed by (A) reducing SDS‐PAGE. A small decrease in mass indicated deglycosylation of the IgGγ chain. (B) LCA displayed significantly reduced binding to plates coated with endoS‐treated h2G7 as compared to nontreated h2G7. (C) EndoS treatment of h2G7 significantly reduced human recombinant CD64. (D) Binding to live THP‐1 cells incubated with PBS‐, h2G7‐, or endoS‐treated h2G7. Results are represented as means ± SEM from three independent experiments. ^***^ *P* < 0.001 by one‐way ANOVA with Bonferroni posttest. Abbreviations: MFI, mean fluorescence intensity; OD, optical density.

Hydrolysis of the N‐linked glycan at position N297 by endoS treatment was verified by a characteristic mass shift (Fig. 6A) and by a reduced binding to LCA (Fig. 6B and Supporting Fig. S5A), which specifically binds to N‐linked glycans. To verify that deglycosylation reduced FcR binding, we evaluated this binding *in vitro*. Human IgG1 has the capacity to bind and activate all members of the FcγR family, and, as predicted, deglycosylation of h2G7 ablated binding to human recombinant FcγRI/CD64 (Fig. 6C and Supporting Fig. S5B). We further investigated binding of h2G7 to human THP‐1 cells that express both CD64 and FcγRII/CD16. The endoS treatment significantly reduced binding of h2G7 to THP‐1 cells (Fig. 6D and Supporting Fig. S5C). The K322A mutant and nonmodified h2G7 displayed similar binding to LCA, CD64, and THP‐1 cells (data not shown).

### THERAPEUTIC PROPERTIES OF EFFECTOR FUNCTION‐DEFICIENT h2G7

**Figure 7:**
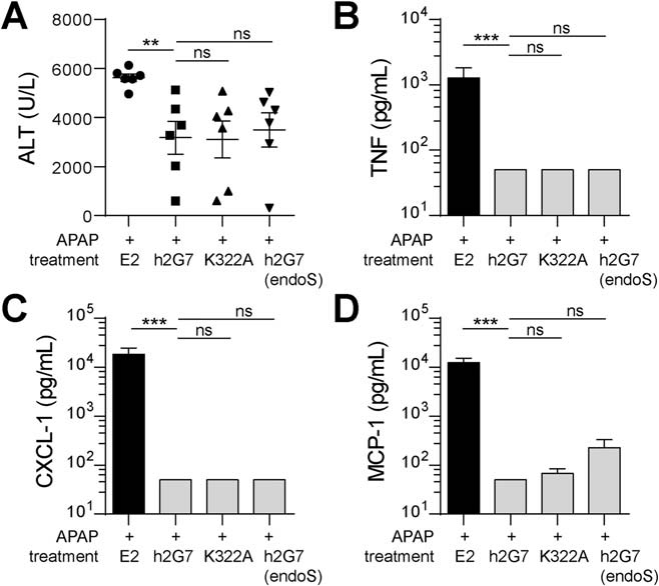
Effector‐function deficient h2G7 variants demonstrate analogous therapeutic effects as h2G7. APAP‐exposed C57BL/6J mice (530mg/kg) were treated with 300μg E2, K322A, endoS‐treated h2G7 or h2G7 at 2h post‐APAP (n = 6). (A) K322A, endoS‐treated h2G7 and h2G7 demonstrate equivalent hepato‐protective effects as measured by serum ALT. No significant difference was noted between the h2G7 treatment groups (gray bars) with respect to anti‐inflammatory activity as measured by a decrease in (B) TNF, (C) CXCL‐1, or (D) MCP‐1. Undetectable levels were substituted with the value for lowest limit of detection (50pg/mL). Data are presented as means ± SEM. ^**^ *P* < 0.01 and ^***^ *P* < 0.001 by Kruskal‐Wallis with Dunn's posttest. Abbreviation: ns, not significant.

We next studied the therapeutic *in vivo* efficacy of the effector function‐deficient h2G7 variants in order to elucidate the mechanism by which h2G7 elicits its hepatoprotective and anti‐inflammatory effect. Treatment with the modified variant antibodies (K322A‐ and endoS‐treated h2G7) and h2G7 in APAP‐challenged mice showed similar hepatoprotective effects, as determined by a comparable reduction of serum ALT (Fig. 7A) and miR‐122 (Supporting Fig. S6). Equivalent anti‐inflammatory effects, as measured by reduced levels of TNF (Fig. 7B), CXCL‐1 (Fig. 7C), and MCP‐1 (Fig. 7D), were also recorded. These results collectively establish that the therapeutic effects of h2G7 were mediated by HMGB1 neutralization rather than by complement activation or Fc receptor‐mediated effects.

## Discussion

APAP is one of the most common over‐the‐counter drugs and is generally safe at recommended therapeutic doses. Nevertheless, APAP intoxication is a clinical problem and may result in ALI or even death. During clinical APAP overdose, a worse prognosis is correlated with activation of postinjury inflammation and systemic inflammatory response syndrome.^2^, ^4^, ^27^, ^33^ Given the inadequacy of current therapy (i.e., NAC), especially in late‐presenting patients, there is a need for development of novel therapies targeting the postinjury inflammation. Specific immune‐modulatory therapies are, as of yet, not available as treatment options for APAP‐ALI, although such therapeutic strategies would potentially prolong the therapeutic window. Indeed, our data strongly support that anti‐HMGB1 therapy significantly prolongs the therapeutic window of experimental APAP‐ALI (Fig. 4) and would likely be invaluable in treatment of late‐presenting patients or in patients that do not respond satisfactorily to NAC. HMGB1 has been shown to be a pivotal regulator and biomarker of injury as a result of APAP overdose.^2^, ^10^ Therapeutic neutralization of HMGB1 has the potential to improve outcome of several human diseases, but no published clinical trials using HMGB1‐specific inhibitors have so far been conducted. A paucity of HMGB1 antagonists suitable for the clinic has, until now, been the major restricting element for further progress. Accordingly, the main purpose of this study was to engineer a chimeric HMGB1‐specific mAb with the potential to be evaluated in future clinical trials, by investigating its therapeutic efficacy in a previously shown HMGB1‐dependent experimental model of APAP‐ALI. We believe that the outcome of our study, the partly humanized h2G7, represents a promising candidate for further clinical exploration.

Conserved proteins are poor immunogens, and raising protective therapeutic mAbs that target such proteins is a challenging enterprise.^34^ Hence, generating therapeutically efficient mAbs specific for HMGB1 (99% sequence conservation in mammalians) has proven no exception to this experience. It took several years after the original polyclonal anti‐HMGB1 antibody treatment study^17^ until an anti‐HMGB1 mAb (m2G7) was demonstrated to confer experimental disease protection.^22^, ^35^ Although a few additional mouse mAbs targeting HMGB1 have mediated successful results in preclinical disease models,^36^, ^37^ none has demonstrated comparable universal therapeutic efficacy as the 2G7 mAb that ameliorates diverse inflammatory disease models (Supporting Table S1).

The generated chimeric 2G7 antibody, with its replacement of the constant m2G7 frameworks with human sequences, may possibly meet the drug development requirements for an HMGB1 specific antibody therapy that would be suitable for clinical trials. Humanization processes may alter both specificity and affinity of an antibody,^23^ and for that reason, we investigated whether a change in the constant Ig framework modified the *in vitro* properties of 2G7. Our results indicate that h2G7 did not interact with anti‐mouse IgG antibodies, implicating that removal of the murine constant frameworks significantly reduced immunogenicity (data not shown), but with preserved antigen specificity and affinity (Fig. 1B,C).

The therapeutic efficacy of m2G7 in preventing HMGB1‐induced sterile inflammation has emphasized the clinical potential of 2G7 in several disorders (Supporting Table S2) and especially in hepatic disorders.^7^, ^35^ HMGB1 levels are increased in clinical and experimental APAP‐ALI and surpass ALT as a predictor of clinical outcome post‐APAP intoxication.^2^, ^10^ The central functional role of HMGB1 in APAP‐ALI pathogenesis is highlighted by the fact that hepatocyte‐specific abrogation of *Hmgb1* and anti‐HMGB1 treatment are highly protective in experimental models of APAP‐ALI.^7^, ^14^ In addition, extensive postinjury inflammation negatively correlates with patient outcome (i.e., need for LT or death). Our results, based on therapeutic interventions with h2G7 or m2G7, are in full agreement with this concept and confirm retained functionality of the novel h2G7 antibody. Furthermore, therapeutic intervention with h2G7 is superior to NAC treatment at late time points of experimental APAP‐ALI, possibly providing a novel treatment specifically targeting postinjury inflammation with a prolonged therapeutic window of opportunity at which NAC treatment fails to confer hepatoprotection.

Recent structural HMGB1 studies emphasize that the redox states of its three conserved cysteine residues regulate the receptor‐binding ability and subsequent biological functions. Fully reduced HMGB1 (all‐thiol) acts a chemotactic factor, partially oxidized HMGB1 (disulfide) induces cytokines through TLR4/MD‐2, whereas the fully oxidized HMGB1 (sulfonyl) exerts no demonstrable inflammatory activity.^6^, ^38^, ^39^ All three isoforms are systemically present in APAP‐challenged mice, although at different stages of postinjury inflammation.^6^ HMGB1 isoforms (various redox and acetylated isoforms) are readily detected by 2G7 in immunoblotting analysis (data not shown) or by direct enzyme‐linked immunosorbent assay (ELISA; Fig 1D,E), but we cannot further comment on conceivable binding preferences of the antibodies to any of the HMGB1 isoforms *in vivo*. It has previously been demonstrated that m2G7 inhibits both HMGB1‐induced cytokine production^39^ as well as HMGB1‐induced cell migration *in vitro* (personal communication with Prof. Marco E. Bianchi). Our *in vivo*‐based studies further support that h2G7 and m2G7 inhibits HMGB1‐driven cytokine and chemokine release in experimental APAP‐ALI equally well (Fig. 2F). Likewise, several *in vivo* models of sterile inflammation have demonstrated that m2G7 suppresses both HMGB1‐induced migration and cytokine production (Supporting Table S1). This collectively suggests that 2G7 hampers the effect of both known inflammatory isoforms of HMGB1.

The hepatoprotective effects and elimination of inflammatory mediators in response to 2G7 treatment underline the pathogenic contribution of inflammation in APAP‐ALI, and that APAP‐induced inflammation is highly HMGB1 dependent. A single injection of 3 μg of h2G7 as well as the hepatoprotective dose of 300 μg completely eliminated the studied circulating inflammatory mediators (Fig. 3). Yang et al. demonstrated that treatment with low‐dose m2G7 (5 μg) per mouse at 2 hours and a repeated dose at 7 hours post‐APAP was both hepatoprotective and anti‐inflammatory at 24 hours post‐APAP.^7^ The higher dose required in our study for hepatoprotection could possibly be explained by experimental kinetic differences regarding the postinjury inflammatory response or that a single low‐dose injection of h2G7 is not sufficient to confer hepatoprotection. Nevertheless, the functional similarities of h2G7 and m2G7 in the current study suggest that h2G7 may exert life‐saving effects in APAP‐induced ALI in a clinical setting.

None of the previous preclinical studies involving m2G7 have addressed the mechanism of action of the m2G7 parental antibody. The present generation of a novel chimeric 2G7 antibody (h2G7) allowed us to, in a controlled manner, modify its effector functions and thus study its mechanism of action. In concurrence with published data,^40^ a K322A substitution of h2G7 completely suppressed C1q binding without affecting binding to FcγRs. The aglycosylated h2G7 was incapable of binding neither C1q nor FcγRs. Given that deglycosylation of antibodies may affect binding to C1q, it is important to recognize both isotype‐ and species‐specific differences, especially when conducting studies comparing therapeutic antibodies.^31^, ^32^, ^41^ The binding of aglycosylated mouse IgG2b to C1q is negligible and it is therefore likely that endoS‐treated m2G7 would have the same effect.^42^ Our *in vivo* data collectively suggest that h2G7 acts through antigen neutralization, rather than complement activation or FcγR engagement, given that neither the K322A mutant nor endoS‐treated h2G7 displayed altered therapeutic activity as compared to nonmodified h2G7. The *in vivo* neutralizing effects of 2G7 could possibly be explained by a direct steric blockade of HMGB1‐receptor interaction. As shown for other therapeutic antibodies, it is conceivable that a competition for receptor binding sites is sufficient for the observed HMGB1 neutralization by h2G7 or that allosteric mechanisms induce or suppress conformational changes, thus altering the function of HMGB1.^43^ Further in‐depth studies are required to define whether h2G7 has a preference for certain HMGB1 isoforms and to elucidate the detailed mechanisms for h2G7‐mediated neutralization.

To summarize, we here report the creation of a partly humanized, chimeric mAb targeting HMGB1 with preserved functionality compared to the parental mouse anti‐HMGB1 mAb. We conclude that HMGB1 neutralization was the observed mechanism of action in experimental APAP‐ALI. The h2G7 antibody would likely provide significant advantages in a clinical setting attributed to reduced xenogeneic immune responses and improved pharmacokinetics, as compared to mouse anti‐HMGB1 mAbs, especially in late‐presenting patients and in patients with a need for repeated treatment. These results provide distinct progress in the endeavor to bring an HMGB1‐specific antagonist to further clinical development.

Author names in bold designate shared co‐first authorship.

## Supporting information

Additional Supporting Information may be found at onlinelibrary.wiley.com/doi/10.1002/hep.28736/suppinfo.

Supporting Information
