# Supplementary material for: A novel high mobility group box 1 neutralizing chimeric antibody attenuates drug‐induced liver injury and postinjury inflammation in mice
Source: Hepatology. 2016 Sep 1;64(5):1699–710. doi: 10.1002/hep.28736 (PMC5082559; doi:10.1002/hep.28736)
Supplement: Supplementary file 1 — Supporting Information [file HEP-64-1699-s001.pdf]

Supplementary data to:

A novel HMGB1 neutralizing chimeric antibody attenuates DILI and post-injury inflammation

Peter Lundbäck[1], Jonathan D. Lea[2], Agnieszka Sowinska[1], Lars Ottosson[3], Camilla Melin Fürst[4], Johanna Steen[1], Cecilia Aulin[1], Joanna I. Clarke[2], Anja Kipar[2], Lena Klevenvall[3], Huan Yang[5], Karin Palmblad[3], B. Kevin Park[2], Kevin J. Tracey[5], Anna M. Blom[4], Ulf Andersson[3], Daniel J. Antoine[2,#], and Helena Erlandsson Harris[1,#]

Supplementary methods

Cloning and protein purification

HMGB1, boxA and boxB cloning and production

All PCR reactions were performed by using Accuprime *pfx* (Life-technologies, Carlsbad, CA) according to manufacturer's instructions unless otherwise stated.

HMGB1 cDNA was cloned into a pet28c vector (Novagen) in order to generate c-terminal 6xHIS-tagged proteins. Primers (Eurofins DNA) used used to amplify cDNA fragments of HMGB1 are stated in SI Table2. Restrictions enzymes used for linearization of vector and cleavage of PCR fragment were NcoI and XhoI (Fermentas). Linearized vectors were treated with shrimp alkaline phosphatase before insertion of cleaved PCR fragments and ligation with T4 DNA ligase (Fermentas). Newly generated plasmids were transformed into Oneshot DH5α competent bacteria (Life Technologies) by a 30sec, 42°C heat pulse. Kanamycin was used as antibiotic for plasmid selection. For recombinant protein production, plasmids were transformed into BL-21DE3 bacteria (Life-technologies) and expression was induced by addition of 1mM IPTG for 16h at room temperature. Bacteria were lyzed in buffer A

(imidazole 20mM and 500mM NaCl) by 4x30sec sonication on ice. Lysate was cleared from debris by centrifugation at 15,000g. Recombinant protein purifications were performed on an ÄKTA Explorer 10 system according to manufacturer's instructions and all buffers used were filtered (0.2μm) and degased before use. Briefly, a HIS-TRAP FF column was equilibrated with 5 column volumes of buffer A before injection of bacterial lysate. The column was washed with increasing concentrations of imidazole (up to 80mM) and protein was eluted with 200mM imidazole and 500mM NaCl. High purity fractions were pooled and the final product was >95% pure. Protein preparations were extensively dialyzed against PBS.

Generation of a chimeric anti-HMGB1 antibody

Cloning was performed as previously described [1, 2]. RNA from hybridoma cells producing monoclonal m2G7 antibodies was isolated by RNeasy columns (Qiagen). Superscript (Life-Technologies) was used to synthesize cDNA. IgGγ and IgGκ variable regions were amplified separately starting from 4μL of cDNA as template with primers stated in SI. Table2. Each round of PCR (50 cycles) was 94°C for 30sec, 58°C for 30sec, and 72°C for 45sec. PCR products were purified using QIAquick PCR purification kit (Qiagen) and digested with restriction enzymes stated in SI. Table2 (New England Biolabs). Digested products were ligated using the quick ligase kit (New England Biolabs) into expression vectors containing the human IgGγ and IgGκ constant regions. Constructs were transformed into DH5α (Invitrogen) and propagated plasmids were isolated with NucleoSpin plasmid DNA isolation kit (Macherey-Nagel). IgGγ and IgGκ plasmids were sequenced (Eurofins DNA) to confirm identity and correct reading frames with the original m2G7 variable region. The irrelevant IgG1 isotype control antibody E2 has specificity towards tetanus toxin and was originally published as 1362SF-E02 [2].

Recombinant antibodies were produced by transient transfection of FreeStyle HEK-293F cells (Gibco, Life Technologies). Cells were cultured at 37°C with 8% CO_2 in FreeStyle 293 Expression medium (Gibco, Life Technologies) to a density of 0.6-0.7x10^6 cells/mL. The cells were then transfected with 0.5µg of vector DNA for each antibody chain (heavy and light) per mL of cell culture, using the PEI-Max transfection reagent (Polysciences) dissolved in OPTI-PRO SFM medium (Gibco, Life Technologies). Supernatants were collected at 9-10 days post-transfection and antibodies were purified on HiTrap Protein G HP columns (GE Healthcare) using an ÄKTA Explorer 10 system (GE Healthcare). PBS was used as running buffer and the antibodies were eluted with 0.1M glycine (pH 2.7) and neutralized with a suitable amount of 1M Tris buffer (pH 9.0). Antibodies were extensively purified against PBS and concentrations were determined by Nanodrop ND-1000 (Thermo Scientific), and the purity (>98%) of the expressed antibodies was determined by SDS-PAGE .

Generation of K322A mutant

An 18-cycle PCR reaction was performed with h2G7 IgGγ plasmid as template with primers stated in SI. Table2. After PCR-cycling, template DNA was digested by DpnI digestion for 1h at 37°C. PCR produced plasmids were purified by gel extraction and sequenced in order to verify the expected codon change (AAG/GCG).

Antibody specificity testing

1µg HMGB1, box A or box B was coated in an ELISA microtiter plate (Nunc maxisorp) at room temperature overnight. Plates were blocked in 1% BSA in PBS (pH 7.4) for 1h at room temperature. Wash steps were performed with 3x PBS-Tween (0.05%). h2G7, m2G7, K322 or E2 was diluted (100, 10, 1 and 0.1 ng/mL) in antibody diluent (0.1% BSA, 0.05% Tween-20 in tris-buffered saline, pH 7.4) and

incubated at room temperature for 2h. Anti-human or anti-mouse IgG was diluted to 1:10000 in antibody diluent and incubated for 1h at room temperature. 3,3',5,5'-tetramethylbenzidine (TMB) substrate was added and stopped after 5min with 2N sulphuric acid. Optical density at 450nm was determined (with subtraction of plate blank).

Surface plasmon resonance (SPR) kinetic experiments

Kinetic experiments were performed on a Biacore 2000 system (GE Healthcare, Uppsala, Sweden) according to manufacturer's instructions unless otherwise stated. All buffers and samples were 0.2μM filtered and degased prior to use and the Biacore system was primed with running buffer (PBS-T 0.01%). **Immobilization**; Immobilization pH scouting was performed for the h2G7 antibody and the identified optimal settings were adapted for all antibodies. Antibodies were diluted in 10mM sodium acetate, pH 5.0, and immobilized on a CM5 sensor chip by amine coupling using *N*-hydroxysuccimide (NHS) and N-ethyl-N-(3-dimethylaminopropyl)-carbodiimide hydrochloride (EDC). Target level for ligand immobilization (R_L) was calculated and set to 600 in order to achieve a theoretical R_{max} of 200. Flow channel 1 (Fc1) was used as reference surface (blank), Fc2 was immobilized with E2, Fc3 with h2G7 and Fc4 with m2G7 antibody. Reactive esters that were not cross-linked were blocked with ethanolamine and 50mM NaOH was used as wash buffer after immobilization. **Kinetic analysis**; A two-fold serial dilution of the analyte HMGB1 was performed (880nM, 440nM, 220nM, 110nM, 55nM and 0nM) in PBS-T. Experiments were performed in direct binding mode at a 30μL/min flow rate at 25ºC and injection time for HMGB1 (sorted low to high concentration) was 3min followed by a dissociation time of 15min before regeneration with two 45sec pulses of regeneration buffer (10mM Glycine-HCl pH 2.0, 1M NaCl). Stabilization time after

every regeneration buffer injection was set to 2min. **Data analysis**; Data was evaluated using BiaEvaluation. Unnecessary data was subtracted and baselines were adjusted to zero. For each HMGB1 concentration injected, the reference surface signal from Fc1 was subtracted from the other channels (i.e. Fc2-Fc1, Fc3-Fc1 and Fc4-Fc1). The binding curves were also blank-run subtracted by removing the 0nM signal for all concentrations. Curve fitting was done by simultaneous Ka/Kd analysis (1:1 Langmuir binding) and a $Chi^2/Rmax < 1\%$ was considered as a good fit.

Redox isoform specificity

1µg disulfide HMGB1 (HMGBiotech, Milano, Italy) was coated for 16h at 4ºC in microtiter plates in 5mM DTT, PBS or 10mM H_2O_2 to generate all-thiol, disulfide or sulphonyl HMGB1. Plates were blocked with 1% BSA for 1h. Increasing concentrations of m2G7 or h2G7 (0.1 – 1000 ng/mL) was added and incubated for 2h at room temperature. Bound antibodies were detected with either HRP-coupled anti-mouse IgG (Sigma-Aldrich, A3854) or anti-human IgG (DAKO Cytomation, Glostrup, Denmark, P0214)

Effector function validations assays

Deglycosylation of h2G7 was performed using deGlycIT columns (Genovis, Lund, Sweden) according to manufacturer's instructions.

Gelshift assay; 3.75µg antibody was subjected to SDS-PAGE on tris glycine 4-20% gradient gels (BioRad). Gel was stained with Coomassie blue and destained in 10% acetic acid and 40% methanol. Deglycosylation effect was verified by a small mass-shift decrease for IgGγ.

***Lens Culinaris* Agglutinin (LCA) binding assay**; Half-area microtiter ELISA plates (Corning) were coated with antibodies at 37°C for 2h. Between each step after coating, plates were thoroughly washed 5x with wash buffer (0.1% TBS-T) and

blocked in wash buffer for 1h at room temperature. Equilibration of plates were performed by 5x washes with TC buffer (1mM Tris pH 7.5, 1mM $CaCl_2$, $MgCl_2$, MnCl2 and 0.1% Tween). Biotinylated LCA (Vector Labs) was diluted to 1μg/mL in TC buffer and added to plates for 1h at 37°C. Streptavidin-HRP was diluted in 0.1% TBS-T and incubated for 20min at room temperature. The reaction was started by addition of TMB substrate solution and stopped with 2N sulphuric acid.

CD64 binding assay; Human recombinant FcγRI/CD64 (Life Technologies) was diluted to 1μg/mL in PBS and coated in a half-area microtiter ELISA plates (Corning) at 37°C for 2h. Plate was washed 3x with PBS-T (0.05%) between each of the following step. Plate was blocked in 1% BSA in PBS for 1h at room temperature. Antibodies were diluted in antibody diluent (0.1% BSA, 0.05% Tween-20 in tris-buffered saline) and added to the plate for 1.5h at room temperature. Rabbit F(ab')2 anti-human (DAKO Cytomation, P0406) was diluted 1:800 in antibody diluent and incubated for 45min at room temperature. Reaction was started by addition of TMB and stopped with 2N sulphuric acid.

THP-1 binding assay; THP-1 cells were incubated with antibodies at different concentrations for 1h at room-temperature. Bound antibodies were visualized by addition of a rabbit F(ab')2 anti-human (DAKO Cytomation, F0315) as recommended by manufacturer. All cell and antibody dilutions were made in PBS supplemented with 2% FBS. Mean fluorescence intensity was determined by a Gallios flow cytometer (Beckman-Coulter). Data was analyzed by Kaluza Analysis Software.

Activation of complement by antibodies specific to HMGB1; Microtiter plates (Maxisorp, Nunc) were coated with 5μg/mL of antibodies and controls (human serum albumin (HSA) and aggregated human IgG), diluted in PBS for 2h at 37°C. Between each incubation step, wells were washed 4x with immunowash (50mM Tris-HCl pH

8.0, 150mM NaCl, 0.1% Tween-20). After blocking the wells with 1% BSA in PBS for 1h at 37°C, wells were incubated with increasing amounts of normal human serum (NHS) diluted in GVB^{2+} (5mM veronal buffer pH 7.4, 144mM NaCl, 1mM $MgCl_2$, 0.15mM $CaCl_2$, and 1% gelatin) for 45min at 37°C. Deposited C1q was detected with specific antibodies (DAKO, A0136) followed by HRP-conjugated secondary antibodies (DAKO, P0399). The plates were developed with o-phenylenediamine (OPD) substrate (DAKO) and H_2O_2 and the absorbance at 490 nm was measured.

Binding of specific antibodies to HMGB1 and activation of complement; Microtiter plates (Maxisorp, Nunc) were coated with 5µg/mL HMGB1 in PBS, overnight at 4°C. After blocking the wells with 1% BSA in PBS for 1h at 37°C, wells were incubated with 5µg/mL of antibodies and controls diluted in PBS for 1h at room temperature. Increasing amounts of NHS diluted in GVB^{2+} were added and incubated for 45min at 37°C. Deposited C1q was then detected as described previously.

Animal procedures

The protocols were approved and performed in accordance with outlines in a license granted under the Animals (Scientific Procedures) Act 1986 and approved by the University of Liverpool Animal Ethics Committee (United Kingdom) or by the north ethical committee in Stockholm (Sweden). Eight week old male C57BL/6J or CD-1 mice (20-25g) were purchased from Charles River laboratories and had a 7-day acclimatization period in a 12h light/dark cycle and food and water was given *ad libitum* prior to experiments.

Mice (n = 6 or 10 for C57BL/6 or n = 5 or 7 for CD-1 mice) were fasted overnight for 15-16h. SI Figure1. Animals were challenged with an intraperitoneal (IP) injection of APAP (530mg/kg or 300mg/kg when indicated) or vehicle (0.9% saline). At 2h post-APAP (or 6h when indicated), mice were given 300µg of indicated

antibody (or as stated), 500mg/kg NAC or an equal volume PBS. At 10h post-APAP (or 24h when indicated), mice were sacrificed using CO_2 and blood was taken by cardiac puncture. Serum was isolated by centrifugation at 1500*g* for 10min at 4°C. Livers were snap frozen in liquid nitrogen and stored at -80°C, or fixed in 4% PFA overnight at 4°C.

Histological determination of hepatotoxicity

Fixed liver sections were embedded in paraffin wax and 3µm sections were prepared and stained with hematoxylin and eosin (H&E) or Periodic Acid Schiff (PAS) stain. All sections were examined and the degree of hepatotoxicity was scored according previously used criteria [3]. All examination and scoring of sections was performed by Prof. A. Kipar, in a blinded manner.

Immuno-histochemistry stainings

Liver sections were deparaffinized in xylene and rehydrated with ethanol. Subsequently, for antigen retrieval the slides were thermally processed in citrate buffer (pH 6.0) using Retriever2100 (Electron Microscopy Sciences, Hatfield, PA, USA) prior to immunostaining. To block endogenous peroxidase activity, sections were treated with 1% H_2O_2 followed by a blocking buffer provided by the Histostain kit (Life Technologies) followed by an avidin, biotin blocking step (Vector Laboratories Inc., Burlingame, USA). The slides were thereafter incubated over night with a rabbit anti-HMGB1 antibody (5µg/ml, ab18256, Abcam, Cambridge, MA, USA) or rabbit anti-Ki67 antibody (abcam ab16667, diluted 1/100). For HMGB1, immunochemical reactions were developed using the Histostain Plus 3-amino-9-ethylcarbazol (AEC) detection system (Life Technologies) and Ki67 was visualized using Bright Vision Ultimate DAB system (Immunologic, Duiven, Netherlands) before counterstained with hematoxylin. In each assay, a primary rabbit

immunoglobulin of irrelevant antigen-specificity was included as a negative control (Negative rabbit control, DAKO). Assessment of proliferation in the liver was performed by counting the number of Ki67 positive cells per mm2 using Leica QWin V3 tips cell count image analysis program. In total, 7 fields per liver section were analyzed in 6 individual mice for m2G7, h2G7 and PBS. For E2 n=5.

Determination of total hepatic glutathione (GSH)

Total hepatic GSH was determined as described previously [4]. Briefly, 30-50mg of liver was homogenized in 800µL of GSH stock buffer (143mM NaH_2PO_4, 6.3mM EDTA, pH 7.4) supplemented with 200µL of 6.5% (w/v) SSA and incubated on ice for 10min in order to deproteinize samples. Homogenates were centrifuged (18400*g*, 5min, 4°C) and the supernatants stored at -80°C. The protein pellets were dissolved in 1M NaOH at 60°C for 1h and protein concentration of the dissolved pellet was determined using Bio-Rad protein assay (Bio-Rad Laboratories, Hemel Hempstead, UK). GSH content of the supernatants was measured by kinetic reaction (at 412nm) as previously described [5]. GSH in samples was compared to a 0 – 40 nmoles/mL standard curve and all samples were normalized to protein content.

Determination of alanine aminotransferase (ALT) activity in serum

Serum ALT activity was determined by kinetic assay according to the manufacturer's instructions (Thermo Scientific). 30µL of serum was loaded in duplicate into 96-well plates. ALT reagent was heated to 37°C and 300µL per well was added to samples and assayed at 340nm.

MicroRNA 122 (miR-122) quantification in serum

miR-122 was quantified in serum as previously described [6]. Total miRNA was extracted and purified using a miRNeasy kit followed by an RNeasy MinElute

Cleanup Kit (Qiagen, Venlo, Netherlands), in accordance with the manufacturer's instructions. The RNA was eluted in 14µL of nuclease-free water before storing at -80°C until further use.

Reverse transcription was performed using a TaqMan miRNA reverse transcription kit (Applied Biosystems) and miR-122 and Let-7d (endogenous miRNA control) primers. Briefly, 2µL purified miRNA was used to synthesise cDNA with a total reaction volume of 15µL via thermal cycling (30min at 16°C, 30min at 42°C, 5min at 85°C and then held at 4°C). Quantitative-PCR (qPCR) reactions were run in duplicate in 384-well plates using TaqMan PCR Primers and Master Mix (Applied Biosystems) according to manufacturer's instructions. 1.33µL of cDNA was used and the total reaction volume was made up to 20µL with primer/master mix, and subject to thermal cycling (2min at 50°C, 10min at 95°C and 50 cycles of 15sec at 95°C and 60sec at 60°C). miR-122 levels were subsequently normalized to the level of let-7d.

Chemo- and cytokine quantification in serum

Serum MCP-1, CXCL-1, TNF, IFNγ, IL-1β and IL-6 concentration was determined by cytokine bead array (CBA) according to the manufacturer's instructions (BD Biosciences).

Statistical analysis

For *in vivo* studies, Kruskal-Wallis with Dunns post-test was performed. For *in vitro* data, two-tailed *t*-test was performed where indicated. All statistical analysis was performed using Graphpad Prism.

Supplementary Table 1.

Beneficial effects of m2G7 treatment in diverse experimental models

Year	Author	Effects of m2G7 treatment	Associated disease	
2005	Tsung A, *et al*. [7]	Reduced liver injury and inflammation	Ischemia / Reperfusion injury	**Sterile inflammation**
2010	Gao Q, *et al*. [8]	Reduced inflammation and improved pancreatic islet viability	Organ transplantation	
2011	Schierbeck H, *et al*. [9]	Reduced clinical signs of arthritis	Arthritis	
2014	Agalave NM, *et al*. [10]	Reduced pain hypersensitivity	Arthritis	
2014	Parker KH, *et al.* [11]	Reduced number of myeloid-derived suppressor cells in tumors	Cancer	
2015	Yang H, *et al.* [12]	Improved survival, reduced liver injury and inflammation	Acetaminophen-induced liver injury	
2006	Qin S, *et al.* [13]	Improved survival	Sepsis	**Infectious**
2012	Entezari M, *et al*. [14]	Protected against neutrophil recruitment, lung injury and bacterial infection	Pulmonary infection with *P.aeruginosa*	
2012	Chavan SS, *et al.* [15]	Reduced cognitive impairment	Sepsis	
2013	Achouiti A, *et al*. [16]	Reduced lung pathology and inflammation	Pneumonia	
2013	Patel VS, *et al.* [17]	Reduced bacterial counts and lung injury	Respiratory distress/ hypoxia	
2013	Valdés-Ferrer SI, *et al*. [18]	Reduced splenomegaly and inflammation	Sepsis	

Supplementary Table 2.

Cloning primes

use		sequence (5' – 3')	restriction site
IgGγ	forward	CTGCAACCGGTGTACATTCCCAGGTGCAGCTGCAGCAGTCTGG	AgeI
	reverse	TGCGAAGTCGACGCTGCAGAGACAGTGACCAGAG	SalI
IgGκ	forward	CTGCAACCGGTGTACATTCCGAAAATGTTCTCACCCAGTCTCCA	AgeI
	reverse	GCCACCGTACGTTTTATTTCCAGCTTGGTC	BsiWI
HMGB1-HIS	forward	GCGCCATGGGCAAAGGAGATCCTAAGA	NcoI
	reverse	GCGCTCGAGTTCATCATCATCATCTTCT	XhoI
box A-HIS	forward	GCGCCATGGGCAAAGGAGATCCTAAGA	NcoI
	reverse	GCGCTCGAGGAACTTCTTTTTTGTCTCC	XhoI
box B-HIS	forward	AAGCCATGGGCAAGGATCCCAATGCAC	NcoI
	reverse	GCGCTCGAGGACAACTCCCTTTTTTGCT	XhoI
K322A	forward	CAAGGAGTACAAGTGCGCGGTCTCCAACAAAGC	
	reverse	GCTTTGTTGGAGACCGCGCACTTGTACTCCTTG	

Supplementary Fig. 1

Schematic *in vivo* experimental overview. Mice were fasted for 15-16h before challenge IP with APAP (530mg/kg). At 2h post-APAP mice were treated with either PBS (vehicle) or 300µg of indicated antibodies. Mice were euthanized at 10h post-APAP. Serum ALT and miR-122 were measured as markers of hepatotoxicity and serum TNF, CXCL-1 and MCP-1 were used as markers of inflammation. Livers were harvested for histological analysis and to determine hepatic glutathione (GSH).

Supplementary Fig. 2.

Representative liver from normal C57BL/6 mice stained with hematoxylin and eosin (H&E, upper left) or for HMGB1 expression (upper right). C57BL/6 mice exposed with APAP (530mg/kg) for 10h were stained were stained with either H&E (lower left) or for HMGB1 expression (lower right).

Supplementary Fig. 3.

2G7 treatment reduces hepatocyte proliferation in APAP-challenged mice. C57BL/6 mice were challenged with APAP (530mg/kg) for 10h. At 2h post-APAP, mice were treated with either PBS (vehicle) or 300µg of indicated antibodies. The effect on hepatocyte proliferation was evaluated by Ki67 staining (n=6 for PBS, m2G7 and h2G7 and n=5 for E2 treated mice).

Supplementary Fig. 4.

2G7 or box A treatments do not affect baseline hepatic glutathione (GSH)(n = 5) or APAP-induced GSH depletion (n = 7) in CD-1 mice. Results are represented as means±SEM.

Supplementary Fig. 5.

Effects of endoS treatment of h2G7. EndoS treatment of h2G7 dose-dependently affects its binding to (A) LCA, (B) human CD64 and (C) live THP-1 cells.

Supplementary Fig. 6.

Effector function deficient h2G7 variants do not affect serum miR-122 expression in APAP exposed C57BL/6 mice (n = 6). Results are represented as means±SEM.

Supplementary references

[1] Tiller T, Meffre E, Yurasov S, Tsuiji M, Nussenzweig MC, Wardemann H. Efficient generation of monoclonal antibodies from single human B cells by single cell RT-PCR and expression vector cloning. J Immunol Methods 2008;329:112-124.

[2] Amara K, Steen J, Murray F, Morbach H, Fernandez-Rodriguez BM, Joshua V, et al. Monoclonal IgG antibodies generated from joint-derived B cells of RA patients have a strong bias toward citrullinated autoantigen recognition. J Exp Med 2013;210:445-455.

[3] Antoine DJ, Williams DP, Kipar A, Jenkins RE, Regan SL, Sathish JG, et al. High-mobility group box-1 protein and keratin-18, circulating serum proteins informative of acetaminophen-induced necrosis and apoptosis in vivo. Toxicol Sci 2009;112:521-531.

[4] Williams DP, Antoine DJ, Butler PJ, Jones R, Randle L, Payne A, et al. The metabolism and toxicity of furosemide in the Wistar rat and CD-1 mouse: a chemical and biochemical definition of the toxicophore. J Pharmacol Exp Ther 2007;322:1208-1220.

[5] Vandeputte C, Guizon I, Genestie-Denis I, Vannier B, Lorenzon G. A microtiter plate assay for total glutathione and glutathione disulfide contents in cultured/isolated cells: performance study of a new miniaturized protocol. Cell Biol Toxicol 1994;10:415-421.

[6] Antoine DJ, Dear JW, Lewis PS, Platt V, Coyle J, Masson M, et al. Mechanistic biomarkers provide early and sensitive detection of acetaminophen-induced acute liver injury at first presentation to hospital. Hepatology 2013;58:777-787.

[7] Tsung A, Sahai R, Tanaka H, Nakao A, Fink MP, Lotze MT, et al. The nuclear factor HMGB1 mediates hepatic injury after murine liver ischemia-reperfusion. J Exp Med 2005;201:1135-1143.

[8] Gao Q, Ma LL, Gao X, Yan W, Williams P, Yin DP. TLR4 mediates early graft failure after intraportal islet transplantation. Am J Transplant 2010;10:1588-1596.

[9] Schierbeck H, Lundback P, Palmblad K, Klevenvall L, Erlandsson-Harris H, Andersson U, et al. Monoclonal anti-HMGB1 (high mobility group box chromosomal protein 1) antibody protection in two experimental arthritis models. Mol Med 2011;17:1039-1044.

[10] Agalave NM, Larsson M, Abdelmoaty S, Su J, Baharpoor A, Lundback P, et al. Spinal HMGB1 induces TLR4-mediated long-lasting hypersensitivity and glial activation and regulates pain-like behavior in experimental arthritis. Pain 2014;155:1802-1813.

[11] Parker KH, Sinha P, Horn LA, Clements VK, Yang H, Li J, et al. HMGB1 enhances immune suppression by facilitating the differentiation and suppressive activity of myeloid-derived suppressor cells. Cancer Res 2014;74:5723-5733.

[12] Yang H, Wang H, Ju Z, Ragab AA, Lundback P, Long W, et al. MD-2 is required for disulfide HMGB1-dependent TLR4 signaling. J Exp Med 2015;212:5-14.

[13] Qin S, Wang H, Yuan R, Li H, Ochani M, Ochani K, et al. Role of HMGB1 in apoptosis-mediated sepsis lethality. J Exp Med 2006;203:1637-1642.

[14] Entezari M, Weiss DJ, Sitapara R, Whittaker L, Wargo MJ, Li J, et al. Inhibition of high-mobility group box 1 protein (HMGB1) enhances bacterial clearance and protects against Pseudomonas Aeruginosa pneumonia in cystic fibrosis. Mol Med 2012;18:477-485.
[15] Chavan SS, Huerta PT, Robbiati S, Valdes-Ferrer SI, Ochani M, Dancho M, et al. HMGB1 mediates cognitive impairment in sepsis survivors. Mol Med 2012;18:930-937.
[16] Achouiti A, van der Meer AJ, Florquin S, Yang H, Tracey KJ, van 't Veer C, et al. High-mobility group box 1 and the receptor for advanced glycation end products contribute to lung injury during Staphylococcus aureus pneumonia. Crit Care 2013;17:R296.
[17] Patel VS, Sitapara RA, Gore A, Phan B, Sharma L, Sampat V, et al. High Mobility Group Box-1 mediates hyperoxia-induced impairment of Pseudomonas aeruginosa clearance and inflammatory lung injury in mice. Am J Respir Cell Mol Biol 2013;48:280-287.
[18] Valdes-Ferrer SI, Rosas-Ballina M, Olofsson PS, Lu B, Dancho ME, Ochani M, et al. HMGB1 mediates splenomegaly and expansion of splenic CD11b+ Ly-6C(high) inflammatory monocytes in murine sepsis survivors. J Intern Med 2013;274:381-390.

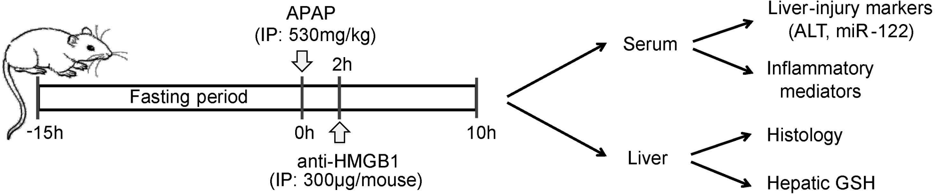
APAP
(IP: 530mg/kg)
2h
Fasting period
-15h
0h
10h
anti-HMGB1
(IP: 300μg/mouse)
Serum
Liver
Liver-injury markers
(ALT, miR-122)
Inflammatory
mediators
Histology
Hepatic GSH

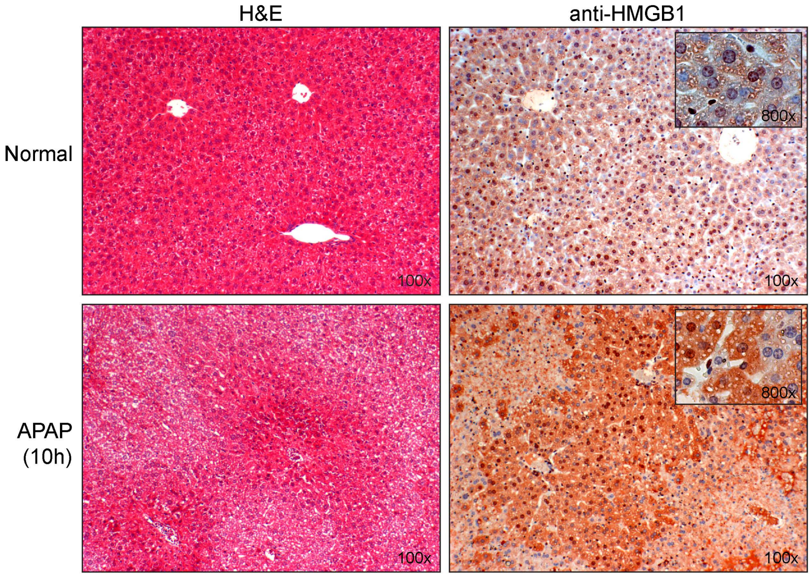
H&E
anti-HMGB1
Normal
APAP
(10h)
100x
800x

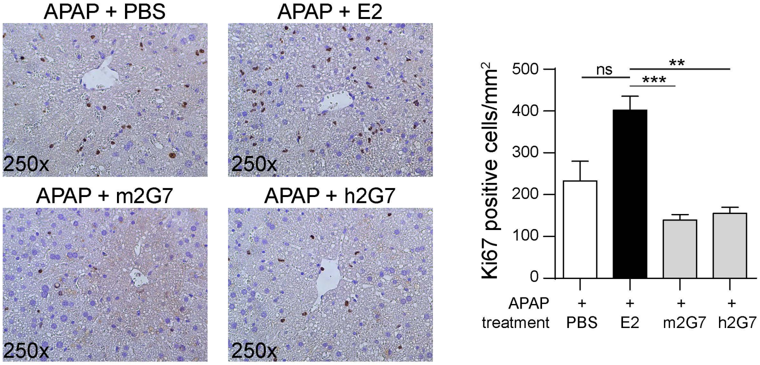
APAP + PBS
APAP + E2
APAP + m2G7
APAP + h2G7
250x
250x
250x
250x
ns
**
***
Ki67 positive cells/mm²
500
400
300
200
100
0
APAP
treatment
+
PBS
+
E2
+
m2G7
+
h2G7

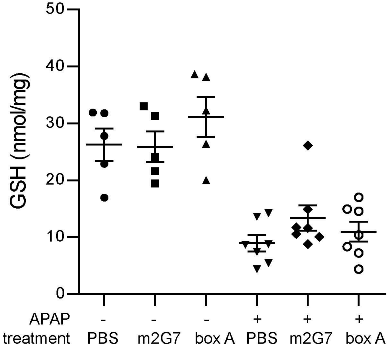

50
40
30
20
10
0
GSH (nmol/mg)
APAP
-
-
-
+
+
+
treatment
PBS
m2G7
box A
PBS
m2G7
box A

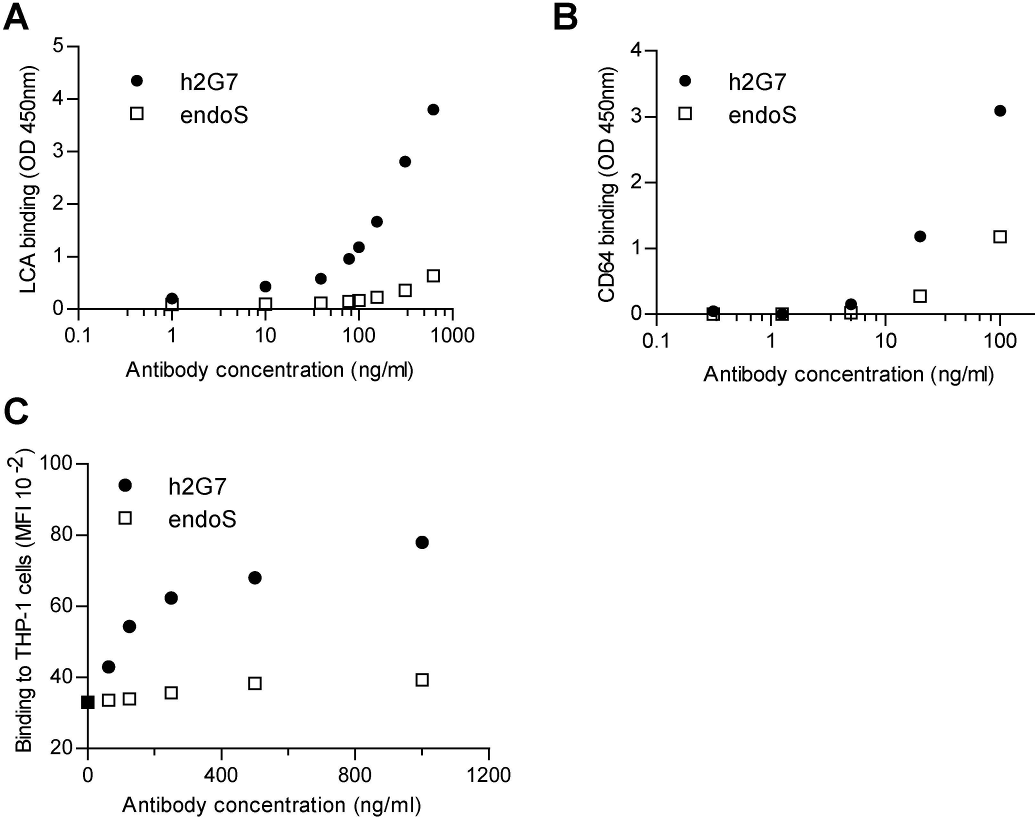

A
LCA binding (OD 450nm)
h2G7
endoS
Antibody concentration (ng/ml)
B
CD64 binding (OD 450nm)
h2G7
endoS
Antibody concentration (ng/ml)
C
Binding to THP-1 cells (MFI 10 -2)
h2G7
endoS
Antibody concentration (ng/ml)

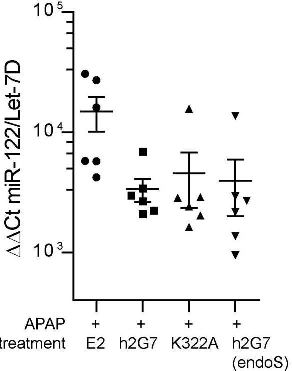

10^5
10^4
10^3
ΔΔCt miR-122/Let-7D
APAP
treatment
+
E2
+
h2G7
+
K322A
+
h2G7
(endoS)
